# Enhancing gadoxetic acid–enhanced liver MRI: a synergistic approach with deep learning CAIPIRINHA-VIBE and optimized fat suppression techniques

**DOI:** 10.1007/s00330-024-10693-9

**Published:** 2024-03-16

**Authors:** Hong Wei, Jeong Hee Yoon, Sun Kyung Jeon, Jae Won Choi, Jihyuk Lee, Jae Hyun Kim, Marcel Dominik Nickel, Bin Song, Ting Duan, Jeong Min Lee

**Affiliations:** 1 Department of Radiology, Seoul National University Hospital, Seoul, 03080 Republic of Korea; 2 Department of Radiology, West China Hospital, Sichuan University, Chengdu, 610041 Sichuan China; 3 Department of Radiology, Seoul National University College of Medicine, Seoul, 03080 Republic of Korea; 4 Department of Radiology, Armed Forces Yangju Hospital, Yangju, 482863 Republic of Korea; 5 MR Application Predevelopment, Siemens Healthcare GmbH, Henkestr. 127, 91052 Erlangen, Germany; 6 Department of Radiology, Sanya People’s Hospital, Sanya, 572000 Hainan China

**Keywords:** Magnetic resonance imaging, Liver, Deep learning, CAIPIRINHA, VIBE

## Abstract

**Objective:**

To investigate whether a deep learning (DL) controlled aliasing in parallel imaging results in higher acceleration (CAIPIRINHA)-volumetric interpolated breath-hold examination (VIBE) technique can improve image quality, lesion conspicuity, and lesion detection compared to a standard CAIPIRINHA-VIBE technique in gadoxetic acid–enhanced liver MRI.

**Methods:**

This retrospective single-center study included 168 patients who underwent gadoxetic acid–enhanced liver MRI at 3 T using both standard CAIPIRINHA-VIBE and DL CAIPIRINHA-VIBE techniques on pre-contrast and hepatobiliary phase (HBP) images. Additionally, high-resolution (HR) DL CAIPIRINHA-VIBE was obtained with 1-mm slice thickness on the HBP. Three abdominal radiologists independently assessed the image quality and lesion conspicuity of pre-contrast and HBP images. Statistical analyses involved the Wilcoxon signed-rank test for image quality assessment and the generalized estimation equation for lesion conspicuity and detection evaluation.

**Results:**

DL and HR-DL CAIPIRINHA-VIBE demonstrated significantly improved overall image quality and reduced artifacts on pre-contrast and HBP images compared to standard CAIPIRINHA-VIBE (*p* < 0.001), with a shorter acquisition time (DL vs standard, 11 s vs 17 s). However, the former presented a more synthetic appearance (both *p* < 0.05). HR-DL CAIPIRINHA-VIBE showed superior lesion conspicuity to standard and DL CAIPIRINHA-VIBE on HBP images (*p* < 0.001). Moreover, HR-DL CAIPIRINHA-VIBE exhibited a significantly higher detection rate of small (< 2 cm) solid focal liver lesions (FLLs) on HBP images compared to standard CAIPIRINHA-VIBE (92.5% vs 87.4%; odds ratio = 1.83; *p* = 0.036).

**Conclusion:**

DL and HR-DL CAIPIRINHA-VIBE achieved superior image quality compared to standard CAIPIRINHA-VIBE. Additionally, HR-DL CAIPIRINHA-VIBE improved the lesion conspicuity and detection of small solid FLLs. DL and HR-DL CAIPIRINHA-VIBE hold the potential clinical utility for gadoxetic acid–enhanced liver MRI.

**Clinical relevance statement:**

DL and HR-DL CAIPIRINHA-VIBE hold promise as potential alternatives to standard CAIPIRINHA-VIBE in routine clinical liver MRI, improving the image quality and lesion conspicuity, enhancing the detection of small (< 2 cm) solid focal liver lesions, and reducing the acquisition time.

**Key Points:**

• *DL and HR-DL CAIPIRINHA-VIBE demonstrated improved overall image quality and reduced artifacts on pre-contrast and HBP images compared to standard CAIPIRINHA-VIBE, in addition to a shorter acquisition time.*

• *DL and HR-DL CAIPIRINHA-VIBE yielded a more synthetic appearance than standard CAIPIRINHA-VIBE.*

• *HR-DL CAIPIRINHA-VIBE showed improved lesion conspicuity than standard CAIPIRINHA-VIBE on HBP images, with a higher detection of small (*< *2 cm) solid focal liver lesions.*

**Supplementary Information:**

The online version contains supplementary material available at 10.1007/s00330-024-10693-9.

## Introduction

Liver magnetic resonance imaging (MRI), including dynamic imaging, is widely used for the detection and characterization of focal liver diseases, and generally demonstrates improved diagnostic performance compared to computed tomography (CT) [1, 2]. Breath-hold three-dimensional (3D) T1-weighted fat-suppressed gradient-recalled echo (GRE) sequences are the cornerstones of multiphasic liver MRI [3], with an acquisition time of 17–23 s in routine clinical practice. Lesion conspicuity is a key determinant for the detection of focal liver lesions, particularly malignant lesions such as hepatocellular carcinoma (HCC) or metastasis [4–6]. The detection of subcentimeter liver malignancies through imaging techniques continues to be challenging, primarily due to the low signal-noise ratio (SNR) in small lesions, limited spatial resolution, and the relatively low contrast caused by their atypical enhancement patterns [7, 8]. Although MRI provides better soft tissue contrast than CT, detection of small lesions on 3D T1-weighted GRE images can be hampered by image degradation owing to motion-related blurring, various artifacts, and the partial volume averaging resulting from the relatively large slice thickness [9, 10]. Given that image quality relies heavily on the breath-hold capability of patients, reducing image acquisition time by rapid imaging techniques is of paramount importance in daily clinical practice [9].

To date, parallel acquisition techniques (PATs) such as sensitivity encoding (SENSE), generalized autocalibrating partially parallel acquisition (GRAPPA), controlled aliasing in parallel imaging results in higher acceleration (CAIPIRINHA), and compressed sensing (CS) have been widely incorporated into the 3D T1-weighted GRE sequences to accelerate imaging acquisition by using under-sampling of the k-space [10–13]. Among these PATs, CAIPIRINHA enables higher acceleration factors by accelerating data acquisition in both the phase encoding and slice encoding directions, leading to shorter acquisition time and reduced image degradation [14]. Previous studies have shown that the CAIPIRINHA volumetric interpolated breath-hold examination (VIBE) provided superior image quality to the GRAPPA [10, 15–17]. However, a high acceleration factor achieved with parallel imaging (PI) or combined PI with CS techniques may cause a g-factor-related SNR loss and aliasing artifacts [9]. Accordingly, these image degradations may also impact the lesion conspicuity and detection [12]. Recently, deep learning (DL) super-resolution (SR) reconstruction algorithms have been shown to reduce examination time and improve image quality and lesion conspicuity in 3D T1-weighted GRE sequences of abdominal MRI [18–21]. To our knowledge, there is currently limited evidence on whether DL-based reconstruction algorithms, in conjunction with optimized spectral fat suppression, can contribute to enhanced image quality and lesion conspicuity in liver MRI.

Therefore, this study aimed to investigate whether a DL CAIPIRINHA-VIBE technique can improve image quality, lesion conspicuity, and lesion detection compared to a standard CAIPIRINHA-VIBE technique in gadoxetic acid–enhanced liver MRI.

## Materials and methods

### Study population

The institutional review board at our institution approved this retrospective study and waived the requirement of written informed consent. We searched the radiologic database of consecutive adult (≥ 18 years) patients who underwent gadoxetic acid–enhanced liver MRI using a 3-T scanner (MAGNETOM Skyra; Siemens Healthcare) between December 20, 2022, and January 20, 2023. Of 180 patients who were initially enrolled, 12 were excluded from the study owing to incomplete liver MR sequences for analysis. Therefore, the final cohort comprised 168 patients (104 men, 64 women; mean age ± standard deviation, 62.1 ± 12.9 years; range 19–88 years). Clinical indications for liver MRI were (a) HCC surveillance (*n* = 120); (b) metastasis surveillance (*n* = 30); and (c) focal liver lesion (FLL) characterization (*n* = 18).

**Fig. 1:**
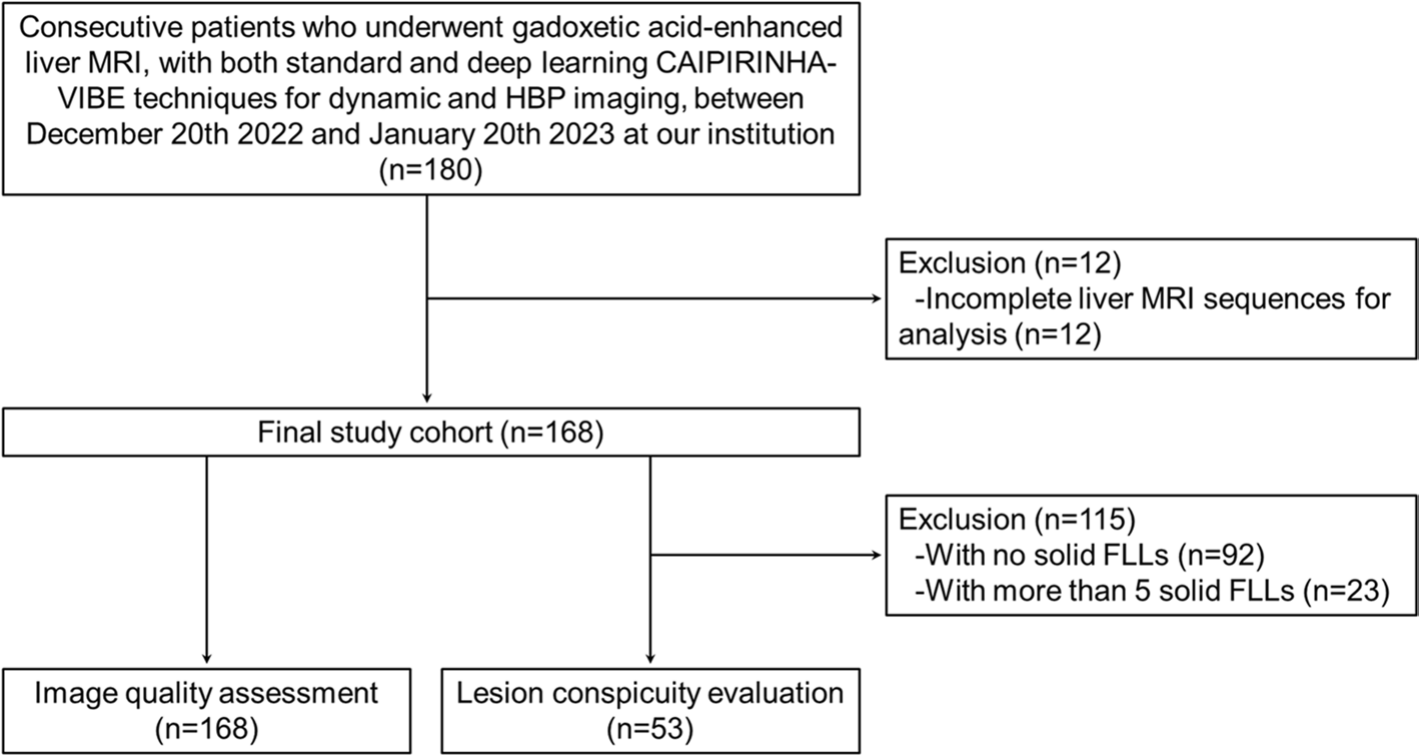
Study flowchart. CAIPIRINHA, controlled aliasing in parallel imaging results in a higher acceleration; FLLs, focal liver lesions; HBP, hepatobiliary phase; MRI, magnetic resonance imaging; VIBE, volume-interpolated breath-hold examination

To ensure a more focused review for solid liver lesions, we focused on patients with ≤ 5 solid FLLs to evaluate lesion conspicuity, excluding nonsolid FLLs, such as treated HCC lesions, benign cysts, typical hemangiomas, and arterioportal shunts. The MRI scans with no solid FLLs (*n* = 92) or more than 5 solid FLLs (*n* = 23) were excluded. Accordingly, 87 solid FLLs detected in 53 patients were included for lesion conspicuity evaluation. The lesion diagnoses were HCCs (*n* = 43), dysplastic nodules (*n* = 17), metastases (*n* = 9), focal nodular hyperplasia (FNH) (*n* = 5), hepatocellular adenomas (*n* = 4), FNH-like lesions (*n* = 2), benign lesions (*n* = 2), inflammatory lesions (*n* = 2), intrahepatic cholangiocarcinoma (*n* = 1), angiomyolipoma (*n* = 1), and sclerosing hemangioma (*n* = 1) (Fig. 1). HCCs were diagnosed based on pathologic examinations or imaging criteria for diagnosing HCC of Korean Liver Cancer Association-National Cancer Center guideline [22]. The diagnoses of metastases were established according to characteristic MRI findings, e.g., irregular or ill-defined margins, rim enhancement on MR dynamic images, hypoenhancement or targetoid appearance on hepatobiliary phase (HBP) images, and exhibiting greater than 20% interval growth on serial cross-sectional imaging in patients with underlying malignancy [23, 24]. Dysplastic nodules were diagnosed based on typical MRI features, like iso- or hyper-intensity on T1-weighted imaging, slight hypointensity on T2-weighted imaging, no arterial phase hyperenhancement (APHE), and isointensity or slight hypointensity on HBP, as well as stability on follow-up cross-sectional imaging [25, 26]. FNHs were diagnosed based on characteristic MRI findings, including homogeneous APHE, central scar, no “washout”, and iso- or hyper-enhancement on HBP, and stable findings on follow-up cross-sectional imaging [24, 27].

### MRI acquisition

MRI examinations were performed on a 3-T scanner (MAGNETOM Skyra; Siemens Healthcare). Routine liver MRI protocols involved T1-weighted dual-echo imaging, pre-contrast and gadoxetic acid–enhanced dynamic and HBP imaging, T2-weighted imaging, and diffusion-weighted imaging using three *b* values (50, 400, and 800 s/mm^2^). A standard dose of 0.025 mmol/kg of contrast agent (Primovist; Bayer Healthcare) was administered at a rate of 1.5 mL/s followed by 25 mL saline flush. For dynamic imaging, triple arterial phase, portal venous phase, transitional phase, and HBP were obtained using a spectrally fat-suppressed 3D VIBE after the injection. The timings for arterial phase (AP) imaging were determined by a real-time bolus-tracking technique with MR fluoroscopic monitoring. All patients underwent the following VIBE protocols: (a) a standard CAIPIRINHA-VIBE scanning and an additional CAIPIRINHA-VIBE with DL reconstruction scanning for pre-contrast images; and (b) a standard CAIPIRINHA-VIBE scanning and an additional CAIPIRINHA-VIBE with DL and high-resolution (HR) DL, respectively, reconstruction scanning for hepatobiliary phase (HBP) images. MRI acquisition parameters are detailed in Table 1. As the HR protocol was acquired in the HBP with more signal, a higher acceleration factor was chosen. The DL CAIPIRINHA acquisitions employed a more efficient sampling scheme for spectral fat suppression. While DL sequences were added to our protocol, they effectively replaced the “standard” CS sequences in practice.

**Table 1:** MRI acquisition parameters

MRI parameter	Standard CAIPIRINHA-VIBE	DL CAIPIRINHA-VIBE	HR-DL CAIPIRINHA-VIBE
Orientation	Axial	Axial	Axial
Repetition time, ms	3.3	3.1	3.1
Echo time, ms	1.21	1.21	1.16
Flip angle, degree	11	11	11
Receiver bandwidth, Hertz/pixel	590	590	600
Field of view, mm^2^	380 × 313	380 × 313	380 × 313
Matrix	352 × 203	352 × 203	320 × 224
Slice thickness, mm	3.0	3.0	1.0
Number of slice	64	64	192
Acquired voxel size, mm^3^	1.1 × 1.5 × 6.0	1.1 × 1.5 × 6.0	1.2 × 1.4 × 2.0
Reconstructed voxel size, mm^3^	0.5 × 0.5 × 3.0	0.5 × 0.5 × 3.0	0.6 × 0.6 × 1.0
Number of excitation	1	1	1
Acceleration factor	4	4	6
Acquisition time, s	17	11	16

*CAIPIRINHA* controlled aliasing in parallel imaging results in higher acceleration, *DL* deep learning, *HR* high resolution, *MRI* magnetic resonance imaging, *VIBE* volumetric interpolated breath-hold examination

For precontrast image, standard CAIPIRINHA-VIBE and DL CAIPIRINHA-VIBE were performed

For HBP image, standard CAIPIRINHA-VIBE, DL CAIPIRINHA-VIBE, and HR-DL CAIPIRINHA-VIBE were performed

### DL reconstruction technique

**Fig. 2:**
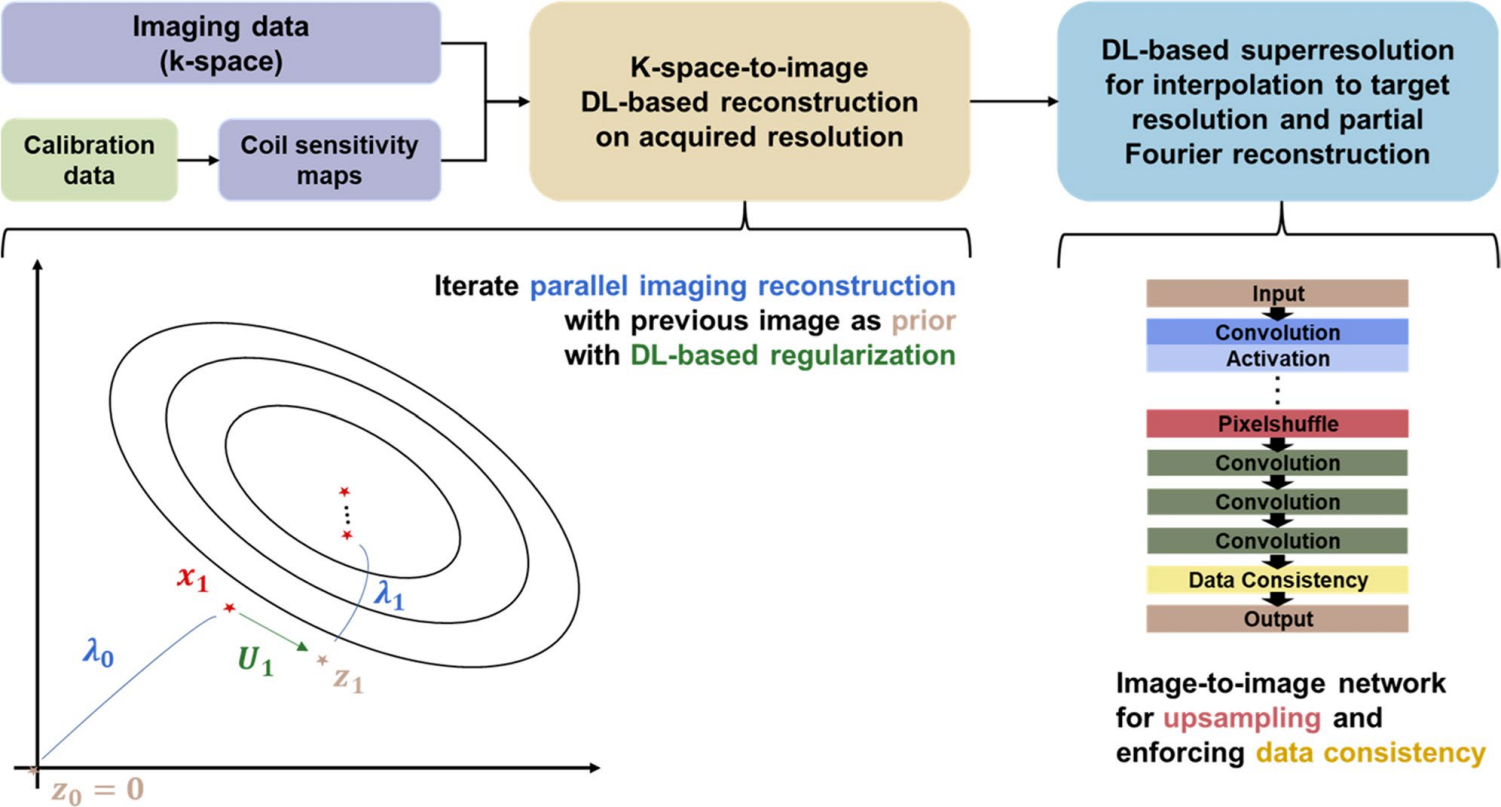
Schematic flow of DL-based reconstruction algorithm. Input and processing steps of the DL-based reconstruction algorithm in the upper row. The lower left diagram illustrates the underlying concept of the k-space to image reconstruction, which alternates between a conventional parallel imaging reconstruction followed by the estimation of a prior image using a neural network U. The conventional reconstruction corresponds to a linear optimization with elliptic hypersurfaces that is pursued from the current prior image with a stepsize λ. The lower right diagram depicts layers of the network architecture used in the super-resolution algorithm. DL, deep learning

The DL-based image reconstruction involved two sequential, independent processing steps (Fig. 2).

In the first step, images were reconstructed from k-space data on the acquired resolution using a network architecture inspired by variational networks [28]. As input, the architecture received undersampled k-space as well as coil sensitivity maps estimated as a preposing step from separately acquired calibration scans. Images were then determined by 6 iterations consisting of a data consistency update in the form of a parallel imaging reconstruction followed by a neural network evaluation for image regularization. Limiting to conventional PAT sampling patterns had the advantage that estimated coil sensitivity maps can be optimized for a given acceleration. Furthermore, as the aliasing in image space was coherent, the training data can be cropped to smaller sizes and thereby allow for a supervised training with image regularization networks acting in all spatial dimensions. The network architecture was implemented in PyTorch [29] and a supervised training performed using about 5000 training pairs derived from about 500 fully sampled 3D datasets acquired from healthy volunteers on 1.5- and 3-T scanners (MAGNETOM scanners, Siemens Healthcare) in the head, abdomen, and pelvis. In alignment with data consistency principles, the network was tailored to enhance local image features, and as with clinically validated 2D methods, it was not expected to be sensitive to the content of the image [30]. A conventional 3D U-net [31] was used for the image regularization networks, and L1 was chosen as loss function and Adam [32] as optimizer. The obtained network was then exported in the ONNX format and integrated into the scanner reconstruction pipeline using the ONNX Runtime [33] as inference engine. Prospective execution time for this processing step was about 15 s for the employed 3D T1-weighted protocols utilizing the scanner integrated graphical processing units.

The second processing step interpolated the acquired images using a DL-based super-resolution algorithm as outlined in prior studies [18, 21]. The employed algorithm performed an initial upsampling by a factor of 2 in all spatial dimensions and was furthermore trained to perform a partial Fourier reconstruction in slice direction, consistent with the chosen acquisition protocol. The dataset utilized for supervised training comprised of high-resolution images, which served as ground-truth images. The input data used in the training were obtained by downsampling these images by a factor of 2 in all spatial dimensions.

Both processing steps were integrated into a research application for prospective use in the scanner reconstruction pipeline.

### Image analysis

All de-identified MR images were independently reviewed by three abdominal radiologists (J.W.C., J.L., and S.K.J.) with 6, 6, and 10 years of experience in abdominal MRI, respectively, who were blinded to the MRI acquisition techniques. The readers underwent a short training session for interpretations and scores of all assessed parameters before initiating image analysis. All MR images of interest (i.e., pre-contrast and HBP images), with either the standard CAIPIRINHA-VIBE or DL CAIPIRINHA-VIBE, and HR-DL CAIPIRINHA-VIBE data sets, were randomly distributed to readers.

#### Qualitative image quality assessment

Image quality was qualitatively evaluated on pre-contrast and HBP axial images, in terms of liver edge sharpness, hepatic vessel conspicuity, bile duct conspicuity (only on HBP image), respiratory motion artifact, cardiac ghosting artifact, ringing artifact, perceived SNR, subjective noise level, synthetic appearance, overall artifact level, and overall image quality on a 4-point scale (Table 2) [13, 34–36]. A higher score implies sharper liver edge, better conspicuity of hepatic vessel and bile duct, less artifact, higher SNR, less noise, less synthetic appearance, and better image quality.

**Table 2:** Scoring criteria for image analysis

Parameter	Score			
	1	2	3	4
Liver edge sharpness Hepatic vessel conspicuity	Not delineated	Moderate blurring	Good delineation	Sharpest border
Bile duct conspicuity	Not delineated	Part or entire biliary system was shown with blurry margin	2nd-order branch was delineated with blurry margin or 1st-order branch was delineated with clear margin	1st- and 2nd-order branches were well delineated with clear margin
Respiratory motion artifact Cardiac ghosting artifact Ringing artifact	Severe artifacts causing impaired diagnostic capability of the readers	Moderate artifacts without diagnostic performance impairment	Mild artifacts without significant image quality disturbance	No or only minimal artifacts
Perceived SNR	Poor SNR (non-diagnostic)	Moderate SNR	Good SNR	Excellent SNR
Subjective noise level	Marked noise level	Moderate noise level	Mild noise level	Negligible noise level
Synthetic appearance	Severe synthetic appearance	Moderate synthetic appearance	Mild synthetic appearance	No synthetic appearance
Overall artifact level	Poor	Fair	Good	Excellent
Overall image quality	Poor	Fair	Good	Excellent
Lesion conspicuity	Poor delineation (not visible)	Fair delineation	Good delineation	Excellent delineation

*SNR* signal–noise ratio

#### Lesion conspicuity and detection evaluation

One researcher (H.W.) with 5 years of experience in abdominal MRI who did not participate in the review session recorded the information of FLLs (i.e., lesion number, size, location, and radiological diagnosis) by reviewing MRI reports and all available clinical information and radiological examinations. All this information was provided to readers for lesion localization. Lesion conspicuity was evaluated on pre-contrast and HBP axial images according to a 4-point scale (Table 2). To exclude nonsolid lesions and treated HCC lesions, matched T2-weighted images and AP images were provided to readers. A higher score indicates better lesion conspicuity.

For lesion detection analysis, lesions with conspicuity scores of 2–4 were defined as detected, while those with conspicuity scores of 1 (not visible) were defined as undetected [34]. Lesion detection rate was calculated by the number of detected solid FLLs divided by the number of total solid FLLs.

### Statistical analysis

Wilcoxon signed-rank test was used for pairwise comparisons of the image quality scores, which were averaged across 3 readers. Interobserver agreement was assessed using the Gwet’s AC1 coefficient [37], as follows: 0.01–0.20, slight agreement; 0.21–0.40, fair agreement; 0.41–0.60, moderate agreement; 0.61–0.80, substantial agreement; and 0.81–1.00, almost perfect agreement. For statistical analyses, the conspicuity scores and number of lesions were considered the sum of observations of 3 readers. Based on the pooled data, lesion conspicuity and detection rate were evaluated by the generalized estimation equation method [38]. Statistical analyses were performed using the R software (version 4.3.1; The R Foundation for Statistical Computing), SAS software (version 9.4; SAS institute), and jackknife free-response receiver operating characteristic software (version 4.2.1). Two-tailed *p* ≤ 0.05 was indicated statistically significant.

## Results

### Qualitative image quality assessment

Comparisons of image quality scores among standard, DL, and HR-DL CAIPIRINHA-VIBE on pre-contrast and HBP images are shown in Table 3.

**Table 3:** Comparisons of image quality scores among standard, DL, and HR-DL CAIPIRINHA-VIBE on precontrast and HBP images

Image quality parameter	Precontrast image				HBP image								
	Standard CAIPIRINHA-VIBE	DL CAIPIRINHA-VIBE	Difference^†^	*p* value	Standard CAIPIRINHA-VIBE	DL CAIPIRINHA-VIBE	HR-DL CAIPIRINHA-VIBE	Difference (DL vs standard)^†^	*p* value	Difference (HR-DL vs standard)^††^	*p* value	Difference (HR-DL vs DL)^†††^	*p* value
Liver edge sharpness	2.71 ± 0.27 (2–4)	3.22 ± 0.25 (1–4)	0.51 (0.46, 0.56)	** < 0.001**	2.85 ± 0.28 (2–4)	3.35 ± 0.22 (1–4)	3.36 ± 0.28 (2–4)	0.51 (0.46, 0.56)	** < 0.001**	0.51 (0.46, 0.56)	** < 0.001**	0.00 (− 0.04, 0.04)	**0.026**
Hepatic vessel conspicuity	2.74 ± 0.34 (2–4)	3.10 ± 0.35 (2–4)	0.36 (0.31, 0.41)	** < 0.001**	2.96 ± 0.33 (1–4)	3.33 ± 0.32 (1–4)	3.42 ± 0.32 (1–4)	0.37 (0.33, 0.41)	** < 0.001**	0.46 (0.42, 0.50)	** < 0.001**	0.09 (0.05, 0.13)	** < 0.001**
Bile duct conspicuity	…	…	…	…	3.02 ± 0.34 (1–4)	3.46 ± 0.29 (1–4)	3.41 ± 0.35 (1–4)	0.44 (0.39, 0.49)	** < 0.001**	0.40 (0.36, 0.44)	** < 0.001**	− 0.04 (− 0.08, 0.00)	0.968
Respiratory motion artifact	3.00 ± 0.36 (1–4)	3.14 ± 0.29 (1–4)	0.14 (0.08, 0.20)	** < 0.001**	3.08 ± 0.32 (1–4)	3.28 ± 0.29 (1–4)	3.28 ± 0.42 (1–4)	0.20 (0.15, 0.25)	** < 0.001**	0.20 (0.14, 0.26)	** < 0.001**	− 0.01 (− 0.06, 0.04)	0.498
Cardiac ghosting artifact	2.85 ± 0.35 (1–4)	3.23 ± 0.25 (2–4)	0.39 (0.33, 0.45)	** < 0.001**	2.94 ± 0.36 (1–4)	3.32 ± 0.27 (2–4)	3.25 ± 0.34 (2–4)	0.38 (0.32, 0.44)	** < 0.001**	0.31 (0.26, 0.36)	** < 0.001**	− 0.07 (− 0.12, − 0.02)	0.240
Ringing artifact	2.88 ± 0.29 (1–4)	3.22 ± 0.26 (2–4)	0.34 (0.29, 0.39)	** < 0.001**	2.98 ± 0.28 (1–4)	3.31 ± 0.27 (1–4)	3.32 ± 0.35 (1–4)	0.33 (0.28, 0.38)	** < 0.001**	0.34 (0.29, 0.39)	** < 0.001**	0.01 (− 0.04, 0.06)	0.080
Perceived SNR	2.67 ± 0.26 (2–4)	2.93 ± 0.31 (2–4)	0.27 (0.22, 0.32)	** < 0.001**	3.09 ± 0.34 (2–4)	3.44 ± 0.34 (2–4)	3.33 ± 0.46 (2–4)	0.35 (0.30, 0.40)	** < 0.001**	0.24 (0.19, 0.29)	** < 0.001**	− 0.11 (− 0.16, − 0.06)	**0.001**
Subjective noise level	2.56 ± 0.34 (1–4)	2.69 ± 0.36 (1–4)	0.13 (0.07, 0.19)	** < 0.001**	2.82 ± 0.32 (1–4)	3.09 ± 0.31 (2–4)	3.12 ± 0.48 (1–4)	0.27 (0.22, 0.32)	** < 0.001**	0.30 (0.24, 0.36)	** < 0.001**	0.03 (− 0.03, 0.09)	0.148
Synthetic appearance	3.37 ± 0.15 (2–4)	2.66 ± 0.30 (2–4)	− 0.71 (− 0.76, − 0.66)	** < 0.001**	3.37 ± 0.15 (2–4)	3.08 ± 0.33 (2–4)	3.36 ± 0.27 (2–4)	− 0.29 (− 0.34, − 0.24)	** < 0.001**	− 0.01 (− 0.05, − 0.03)	**0.018**	0.28 (0.23, 0.33)	** < 0.001**
Overall artifact level	2.66 ± 0.34 (1–4)	2.90 ± 0.29 (1–4)	0.24 (0.19, 0.29)	** < 0.001**	2.78 ± 0.30 (1–4)	3.09 ± 0.30 (1–4)	3.13 ± 0.44 (1–4)	0.30 (0.25, 0.35)	** < 0.001**	0.34 (0.29, 0.39)	** < 0.001**	0.04 (− 0.01, 0.09)	**0.039**
Overall image quality	2.65 ± 0.35 (1–4)	2.83 ± 0.35 (1–4)	0.18 (0.13, 0.23)	** < 0.001**	2.78 ± 0.30 (1–4)	3.16 ± 0.37 (1–4)	3.14 ± 0.48 (1–4)	0.38 (0.33, 0.43)	** < 0.001**	0.37 (0.31, 0.43)	** < 0.001**	− 0.01 (− 0.07, 0.05)	0.747

Unless indicated otherwise, data are means ± standard deviations (ranges). Group comparisons were performed with the paired Wilcoxon signed-rank test

*CAIPIRINHA* controlled aliasing in parallel imaging results in higher acceleration, *DL* deep learning, *HBP* hepatobiliary phase, *HR* high resolution, *SNR* signal–noise ratio, *VIBE* volumetric interpolated breath-hold examination

^†^Data are differences in mean image quality scores between DL CAIPIRINHA-VIBE and standard CAIPIRINHA-VIBE, with 95% confidence intervals in parentheses

^††^Data are differences in mean image quality scores between HR-DL CAIPIRINHA-VIBE and standard CAIPIRINHA-VIBE, with 95% confidence intervals in parentheses

^†††^Data are differences in mean image quality scores between HR-DL CAIPIRINHA-VIBE and DL CAIPIRINHA-VIBE, with 95% confidence intervals in parentheses

#### DL vs standard CAIPIRINHA-VIBE on pre-contrast images

**Fig. 3:**
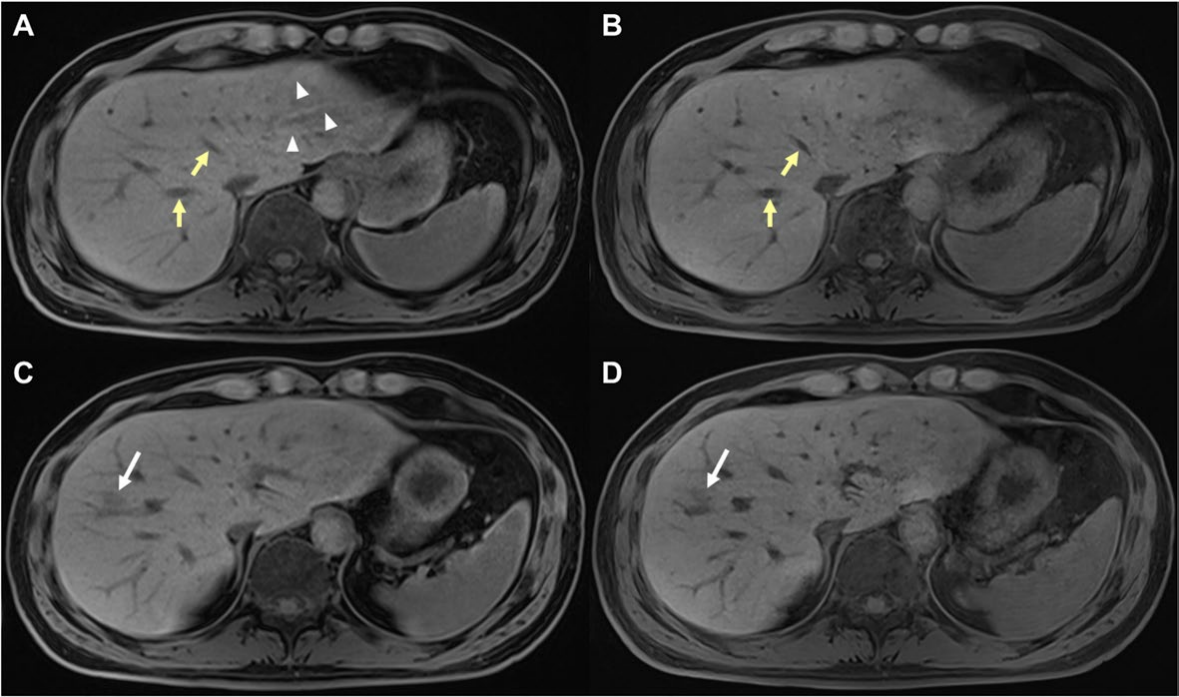
T1-weighted pre-contrast images of gadoxetic acid–enhanced MRI reconstructed with (**A**, **C**) standard CAIPIRINHA-VIBE and (**B**, **D**) DL CAIPIRINHA-VIBE techniques in a 41-year-old male with focal liver lesion (*white arrows*). The (**B**) DL CAIPIRINHA-VIBE shows higher liver edge sharpness, hepatic vessel conspicuity (*yellow arrows*) and perceived SNR, less cardiac ghosting artifact (*arrowheads*) and overall artifact, and better overall image quality than (**A**) standard CAIPIRINHA-VIBE. However, a more synthetic appearance is shown in (**B**) DL CAIPIRINHA-VIBE. The (**D**) DL CAIPIRINHA-VIBE shows lower lesion conspicuity than (**C**) standard CAIPIRINHA-VIBE (mean conspicuity score, 2.33 vs 2.67). CAIPIRINHA, controlled aliasing in parallel imaging results in a higher acceleration; DL, deep learning; MRI, magnetic resonance imaging; SNR, signal–noise ratio; VIBE, volume-interpolated breath-hold examination

On pre-contrast images, DL CAIPIRINHA-VIBE showed significantly higher scores for liver edge sharpness, hepatic vessel conspicuity, respiratory motion artifact, cardiac ghosting artifact, ringing artifact, perceived SNR, subjective noise level, overall artifact level, and overall image quality compared to standard CAIPIRINHA-VIBE (*p* < 0.001 for all). However, the synthetic appearance score of DL CAIPIRINHA-VIBE were significantly lower than standard CAIPIRINHA-VIBE on pre-contrast images (*p* < 0.001) (Fig. 3A, B).

#### DL and HR-DL vs standard CAIPIRINHA-VIBE on HBP images

**Fig. 4:**
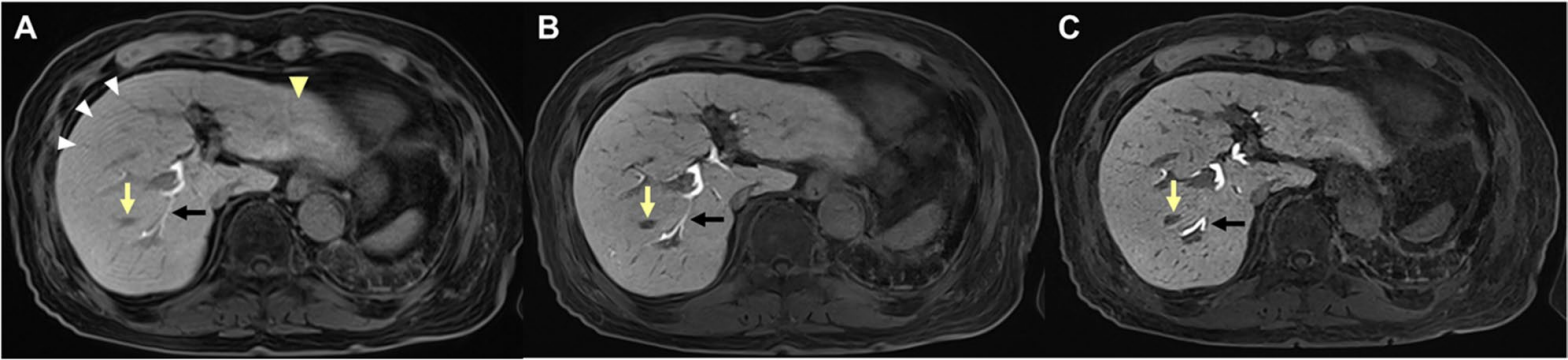
T1-weighted HBP images of gadoxetic acid–enhanced MRI reconstructed with (**A**) standard CAIPIRINHA-VIBE, (**B**) DL CAIPIRINHA-VIBE, and (**C**) HR-DL CAIPIRINHA-VIBE techniques in a 77-year-old male with HCC. The (**B**) DL and (**C**) HR-DL CAIPIRINHA-VIBE show higher liver edge sharpness, hepatic vessel conspicuity (*yellow arrows*), bile duct conspicuity (*black arrows*), less respiratory motion artifact (*white arrowheads*), cardiac ghosting artifact (*yellow arrowhead*) and overall artifact, and better overall image quality than (**A**) standard CAIPIRINHA-VIBE. The (**B**) DL CAIPIRINHA-VIBE shows higher perceived SNR and lower subjective noise level than (**A**) standard CAIPIRINHA-VIBE, while the (**C**) HR-DL CAIPIRINHA-VIBE shows comparable perceived SNR and higher subjective noise level than (**A**) standard CAIPIRINHA-VIBE. However, a more synthetic appearance is shown in both **B** DL and (**C**) HR-DL CAIPIRINHA-VIBE. CAIPIRINHA, controlled aliasing in parallel imaging results in a higher acceleration; DL, deep learning; HBP, hepatobiliary phase; HCC, hepatocellular carcinoma; HR, high resolution; MRI, magnetic resonance imaging; SNR, signal–noise ratio; VIBE, volume-interpolated breath-hold examination

On HBP images, both DL and HR-DL CAIPIRINHA-VIBE demonstrated significantly higher scores for liver edge sharpness, hepatic vessel conspicuity, bile duct conspicuity, respiratory motion artifact, cardiac ghosting artifact, ringing artifact, perceived SNR, subjective noise level, overall artifact level, and overall image quality than standard CAIPIRINHA-VIBE. But the synthetic appearance scores of DL (*p* < 0.001) and HR-DL (*p* = 0.018) CAIPIRINHA-VIBE were significantly lower than standard CAIPIRINHA-VIBE on HBP images (Fig. 4).

#### HR-DL vs DL CAIPIRINHA-VIBE on HBP images

On HBP images, HR-DL CAIPIRINHA-VIBE showed significantly higher scores for liver edge sharpness (*p* = 0.026), hepatic vessel conspicuity (*p* < 0.001), synthetic appearance (*p* < 0.001), and overall artifact level (*p* = 0.039) but lower score of perceived SNR compared to DL CAIPIRINHA-VIBE. No significant differences were detected in bile duct conspicuity, respiratory motion artifact, cardiac ghosting artifact, ringing artifact, subjective noise level, and overall image quality between HR-DL and DL CAIPIRINHA-VIBE on HBP images (*p* ranges, 0.080–0.968) (Fig. 4B, C).

Cases with poorer image quality in DL or HR-DL CAIPIRINHA-VIBE compared to the standard CAIPIRINHA-VIBE on pre-contrast and HBP images are shown in Fig. S1.

#### Interobserver agreement

The interobserver agreement for image quality assessment is summarized in Table 4, with the details provided in Table S1. The Gwet’s AC1 coefficients for liver edge sharpness, hepatic vessel conspicuity, and cardiac ghosting artifact were slight to almost perfect across standard, DL, and HR-DL CAIPIRINHA-VIBE on pre-contrast and HBP images (range, 0.066–0.830); the Gwet’s AC1 coefficients for bile duct conspicuity, perceived SNR, and synthetic appearance were fair to almost perfect (range, 0.234–0.878); the Gwet’s AC1 coefficients for respiratory motion artifact were slight to substantial (range, 0.056–0.769); the Gwet’s AC1 coefficients for ringing artifact and subjective noise level were moderate to almost perfect (range, 0.420–0.841); and the Gwet’s AC1 coefficients for overall artifact level and overall image quality were substantial to almost perfect (range, 0.660–0.936).

**Table 4:** Interobserver agreement for image quality assessment of standard, DL, and HR-DL CAIPIRINHA-VIBE on precontrast and HBP images

Image quality parameter	Precontrast Image		HBP Image		
	Standard CAIPIRINHA-VIBE	DL CAIPIRINHA-VIBE	Standard CAIPIRINHA-VIBE	DL CAIPIRINHA-VIBE	HR-DL CAIPIRINHA-VIBE
Liver edge sharpness	0.349–0.723	0.149–0.770	0.324–0.746	0.136–0.757	0.066–0.812
Hepatic vessel conspicuity	0.428–0.686	0.412–0.762	0.177–0.645	0.327–0.705	0.327–0.830
Bile duct conspicuity	…	…	0.380–0.769	0.357–0.743	0.318–0.824
Respiratory motion artifact	0.106–0.693	0.056–0.631	0.211–0.725	0.117–0.595	0.187–0.769
Cardiac ghosting artifact	0.202–0.728	0.283–0.814	0.205–0.751	0.283–0.774	0.336–0.699
Ringing artifact	0.475–0.801	0.420–0.821	0.579–0.841	0.422–0.833	0.613–0.763
Perceived SNR	0.690–0.841	0.695–0.878	0.480–0.767	0.369–0.743	0.423–0.789
Subjective noise level	0.698–0.749	0.743–0.791	0.726–0.802	0.727–0.838	0.507–0.776
Synthetic appearance	0.460–0.792	0.437–0.825	0.444–0.815	0.286–0.789	0.234–0.685
Overall artifact level	0.782–0.856	0.799–0.908	0.809–0.857	0.799–0.905	0.689–0.763
Overall image quality	0.768–0.818	0.779–0.936	0.754–0.830	0.799–0.920	0.660–0.778

Data are ranges of Gwet’s AC1 coefficients among three readers

Interobserver agreement was assessed by the AC1 coefficients, as follows: 0.01–0.20, slight agreement; 0.21–0.40, fair agreement; 0.41–0.60, moderate agreement; 0.61–0.80, substantial agreement; and 0.81–1.00, almost perfect agreement

*CAIPIRINHA* controlled aliasing in parallel imaging results in higher acceleration, *DL* deep learning, *HBP* hepatobiliary phase, *HR* high resolution, *SNR* signal–noise ratio, *VIBE* volumetric interpolated breath-hold examination

### Lesion conspicuity and detection evaluation

Comparisons of lesion conspicuity scores and detection rates among standard, DL, and HR-DL CAIPIRINHA-VIBE on pre-contrast and HBP images are detailed in Table 5.

**Table 5:** Comparisons of lesion conspicuity and detection rate among standard, DL, and HR-DL CAIPIRINHA-VIBE on precontrast and HBP images

Parameter and group	No		Precontrast image				HBP image								
Lesion conspicuity	Patient	Lesion	Standard CAIPIRINHA-VIBE^§^	DL CAIPIRINHA-VIBE^§^	Difference (DL vs standard)^†^	*p* value^#^	Standard CAIPIRINHA-VIBE^§^	DL CAIPIRINHA-VIBE^§^	HR-DL CAIPIRINHA-VIBE^§^	Difference (DL vs standard)^†^	*p* value^#^	Difference (HR-DL vs standard)^††^	*p* value^##^	Difference (HR-DL vs DL)^†††^	*p* value^###^
All solid FLLs	53	87	1.96 ± 1.09 (1–4)	1.92 ± 1.00 (1–4)	− 0.03 (− 0.14, 0.07)	0.515	2.88 ± 0.89 (1–4)	2.92 ± 0.94 (1–4)	3.20 ± 0.86 (1–4)	0.05 (− 0.04, 0.13)	0.271	0.33 (0.25, 0.40)	** < 0.001**	0.28 (0.17, 0.39)	** < 0.001**
FLLs with size < 2 cm	38	58	1.66 ± 0.98 (1–4)	1.67 ± 0.89 (1–4)	0.01 (− 0.12, 0.14)	0.862	2.63 ± 0.89 (1–4)	2.68 ± 0.96 (1–4)	3.04 ± 0.90 (1–4)	0.05 (− 0.05, 0.15)	0.299	0.41 (0.32, 0.51)	** < 0.001**	0.36 (0.25, 0.48)	** < 0.001**
FLLs with size ≥ 2 cm	26	29	2.55 ± 1.06 (1–4)	2.43 ± 1.03 (1–4)	− 0.13 (− 0.24, − 0.01)	**0.032**	3.38 ± 0.67 (1–4)	3.41 ± 0.66 (1–4)	3.53 ± 0.68 (1–4)	0.03 (− 0.08, 0.15)	0.563	0.15 (0.04, 0.26)	**0.006**	0.11 (− 0.04, 0.27)	0.154
Lesion detection rate	Patient	Lesion	Standard CAIPIRINHA-VIBE^‡^	DL CAIPIRINHA-VIBE^‡^	Odds ratio (DL vs standard)^¶^	*p* value*	Standard CAIPIRINHA-VIBE^‡^	DL CAIPIRINHA-VIBE^‡^	HR-DL CAIPIRINHA-VIBE^‡^	Odds ratio (DL vs standard)^¶^	*p* value*	Odds ratio (HR-DL vs standard)^¶¶^	*p* value**	Odds ratio (HR-DL vs DL)^¶¶¶^	*p* value***
All solid FLLs	53	87	53.6 (140/261)	55.2 (144/261)	1.07 (0.81, 1.41)	0.656	91.2 (238/261)	93.1 (243/261)	94.6 (247/261)	1.34 (0.62, 2.89)	0.454	1.75 (1.03, 2.97)	**0.040**	1.32 (0.64, 2.72)	0.448
FLLs with size < 2 cm	38	58	39.7 (69/174)	44.8 (78/174)	1.22 (0.88, 1.71)	0.238	87.4 (152/174)	89.7 (156/174)	92.5 (161/174)	1.28 (0.60, 2.70)	0.525	1.83 (1.04, 3.23)	**0.036**	1.46 (0.66, 3.21)	0.347
FLLs with size ≥ 2 cm	26	29	81.6 (71/87)	75.9 (66/87)	0.75 (0.59, 0.95)	**0.020**	98.9 (86/87)	100.0 (87/87)	98.9 (86/87)	NA	NA	1.00 (0.08, 12.68)	> 0.99	NA	NA

Using the pooled data of three readers, lesion conspicuity and detection rate were assessed by the generalized estimation equation

*CAIPIRINHA* controlled aliasing in parallel imaging results in higher acceleration, *DL* deep learning, *FLLs* focal liver lesions, *HBP* hepatobiliary phase, *HR* high resolution, *VIBE* volumetric interpolated breath-hold examination

^§^Data are means ± standard deviations (ranges)

^†^Data are differences in mean lesion conspicuity scores between DL CAIPIRINHA-VIBE and standard CAIPIRINHA-VIBE, with 95% confidence intervals in parentheses

^††^Data are differences in mean lesion conspicuity scores between HR-DL CAIPIRINHA-VIBE and standard CAIPIRINHA-VIBE, with 95% confidence intervals in parentheses

^†††^Data are differences in mean lesion conspicuity scores between HR-DL CAIPIRINHA-VIBE and DL CAIPIRINHA-VIBE, with 95% confidence intervals in parentheses

^‡^Data are percentages, with the pooled number of detected lesions divided by the pooled number of total lesions in parentheses

^**¶**^Data are odds ratios between standard CAIPIRINHA-VIBE (reference) and DL CAIPIRINHA-VIBE, with 95% confidence intervals in parentheses

^**¶¶**^Data are odds ratios between standard CAIPIRINHA-VIBE (reference) and HR-DL CAIPIRINHA-VIBE, with 95% confidence intervals in parentheses

^**¶¶¶**^Data are odds ratios between DL CAIPIRINHA-VIBE (reference) and HR-DL CAIPIRINHA-VIBE, with 95% confidence intervals in parentheses

^#^Lesion size adjusted *p* values for the differences in mean scores between standard CAIPIRINHA-VIBE and DL CAIPIRINHA-VIBE

^##^Lesion size adjusted *p* values for the differences in mean scores between standard CAIPIRINHA-VIBE and HR-DL CAIPIRINHA-VIBE

^###^Lesion size adjusted *p* values for the differences in mean scores between DL CAIPIRINHA-VIBE and HR-DL CAIPIRINHA-VIBE

^*^Lesion size adjusted *p* values for the odds ratios between standard CAIPIRINHA-VIBE (reference) and DL CAIPIRINHA-VIBE

^**^Lesion size adjusted *p* values for the odds ratios between standard CAIPIRINHA-VIBE (reference) and HR-DL CAIPIRINHA-VIBE

^***^Lesion size adjusted *p* values for the odds ratios between DL CAIPIRINHA-VIBE (reference) and HR-DL CAIPIRINHA-VIBE

#### DL vs standard CAIPIRINHA-VIBE on pre-contrast images

For all solid FLLs and for FLLs with size < 2 cm, no significant differences were detected in the lesion conspicuity scores between the standard and DL CAIPIRINHA-VIBE on pre-contrast images (*p* = 0.515 and 0.862, respectively). However, for FLLs with size ≥ 2 cm, the standard CAIPIRINHA-VIBE showed significantly higher score for lesion conspicuity compared with the DL CAIPIRINHA-VIBE (2.55 ± 1.06 vs 2.43 ± 1.03; *p* = 0.032). Likewise, for all solid FLLs and for FLLs with size < 2 cm, there were no significant differences in lesion detection rates between the standard and DL CAIPIRINHA-VIBE on pre-contrast images (*p* = 0.656 and 0.238, respectively). However, for FLLs with size ≥ 2 cm, the standard CAIPIRINHA-VIBE showed significantly higher lesion detection rate than the DL CAIPIRINHA-VIBE on pre-contrast images (81.6% [71/87] vs 75.9% [66/87]; odds ratio [OR] = 0.75; *p* = 0.020) (Fig. 3C, D).

#### DL vs standard CAIPIRINHA-VIBE on HBP images

**Fig. 5:**
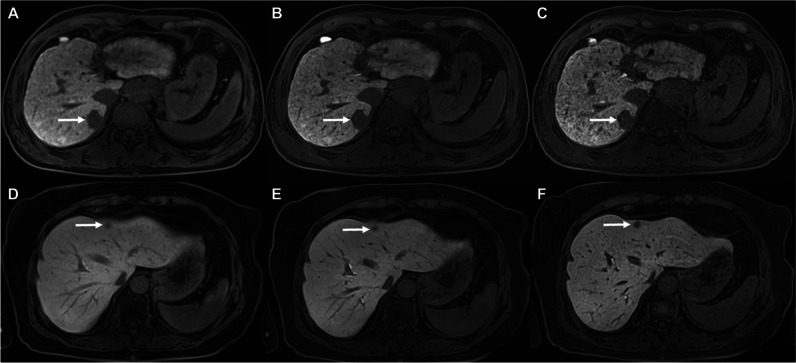
**A**, **B**, **C** T1-weighted HBP images of gadoxetic acid–enhanced MRI in a 69-year-old male with a 2.6 cm HCC (*arrows*) at segment 7 of the liver. The **C** HR DL CAIPIRINHA-VIBE shows higher lesion conspicuity than (**A**) standard CAIPIRINHA-VIBE (mean conspicuity score, 4 vs 3.67), while the **B** DL CAIPIRINHA-VIBE shows comparable lesion conspicuity with (**A**) standard CAIPIRINHA-VIBE (mean conspicuity score, 3.67 vs 3.67). **D**, **E**, **F** T1-weighted HBP images of gadoxetic acid–enhanced MRI in a 76-year-old female with a 1.1-cm metastasis (*arrows*) from lung cancer at segment 4 of the liver. Likewise, the (**F**) HR DL CAIPIRINHA-VIBE demonstrates higher lesion conspicuity than (**D**) standard CAIPIRINHA-VIBE (mean conspicuity score, 3.67 vs 2.67), whereas the **E** DL CAIPIRINHA-VIBE demonstrates lower lesion conspicuity than (**D**) standard CAIPIRINHA-VIBE (mean conspicuity score, 2.33 vs 2.67). CAIPIRINHA, controlled aliasing in parallel imaging results in a higher acceleration; DL, deep learning; HBP, hepatobiliary phase; HCC, hepatocellular carcinoma; HR, high resolution; MRI, magnetic resonance imaging; VIBE, volume-interpolated breath-hold examination

On HBP images, no significant differences were detected in the lesion conspicuity scores between the DL and standard CAIPIRINHA-VIBE for all solid FLLs (2.92 ± 0.94 vs 2.88 ± 0.89; *p* = 0.271), FLLs with size < 2 cm (2.68 ± 0.96 vs 2.63 ± 0.89; *p* = 0.299), and FLLs with size ≥ 2 cm (3.41 ± 0.66 vs 3.38 ± 0.67; *p* = 0.563). Similarly, there were no significant differences in lesion detection rates between the standard and DL CAIPIRINHA-VIBE on HBP images (*p* = 0.454 for all solid FLLs; and *p* = 0.525 for FLLs with size < 2 cm) (Fig. 5A, B, D, E).

#### HR-DL vs standard CAIPIRINHA-VIBE on HBP images

On HBP images, the lesion conspicuity scores of HR-DL CAIPIRINHA-VIBE were significantly higher than standard CAIPIRINHA-VIBE for all lesions (3.20 ± 0.86 vs 2.88 ± 0.89, *p* < 0.001), FLLs with size < 2 cm (3.04 ± 0.90 vs 2.63 ± 0.89, *p* < 0.001), and FLLs with size ≥ 2 cm (3.53 ± 0.68 vs 3.38 ± 0.67, *p* = 0.006). Additionally, the lesion detection rates of HR-DL CAIPIRINHA-VIBE were significantly higher than standard CAIPIRINHA-VIBE for all lesions (94.6% [247/261] vs 91.2% [238/261]; OR = 1.75; *p* = 0.040) and for FLLs with size < 2 cm (92.5% [161/174] vs 87.4% [152/174]; OR = 1.83; *p* = 0.036). However, for FLLs with size ≥ 2 cm, there was no significant difference in the lesion detection rate between the HR-DL and standard CAIPIRINHA-VIBE on HBP images (98.9% [86/87] vs 98.9% [86/87]; OR = 1.00; *p* > 0.99) (Fig. 5A, C, D, F).

#### HR-DL vs DL CAIPIRINHA-VIBE on HBP images

On HBP images, the lesion conspicuity scores of HR-DL CAIPIRINHA-VIBE were significantly higher than DL CAIPIRINHA-VIBE for all lesions (3.20 ± 0.86 vs 2.92 ± 0.94, *p* < 0.001) and for FLLs with size < 2 cm (3.04 ± 0.90 vs 2.68 ± 0.96, *p* < 0.001). Nevertheless, for FLLs with size ≥ 2 cm, no significant difference was detected in lesion conspicuity score between the HR-DL and DL CAIPIRINHA-VIBE on HBP images (3.53 ± 0.68 vs 3.41 ± 0.66, *p* = 0.154). In addition, there were no significant differences in lesion detection rates between the HR-DL and DL CAIPIRINHA-VIBE on HBP images (*p* = 0.448 for all solid FLLs; *p* = 0.347 for FLLs with size < 2 cm) (Fig. 5B, C, E, F).

Cases with lower lesion conspicuity score in DL or HR-DL CAIPIRINHA-VIBE compared to the standard CAIPIRINHA-VIBE on pre-contrast and HBP images are shown in Fig. S2-4.

## Discussion

In this study, DL and HR-DL CAIPIRINHA-VIBE demonstrated improved image quality and reduced artifacts compared to standard CAIPIRINHA-VIBE, with a shorter acquisition time (DL vs standard, 11 s vs 17 s). The reduction in acquisition time, a critical factor for diminishing motion artifacts and enhancing image quality, was principally achieved through the optimization of spectral fat suppression techniques. Yet, the former presented a more synthetic appearance. Moreover, using the DL algorithm for higher spatial resolution within a comparable acquisition time (16 s) to the standard, HR-DL CAIPIRINHA-VIBE notably enhanced lesion conspicuity, especially for small solid FLLs. Our findings are consistent with prior studies on DL-reconstruction algorithms for 3D GRE sequences, underscoring the effectiveness of DL SR reconstruction in abdominal MRI. Afat et al showed that DL SR with partial Fourier acquisition technique enabled improved image quality and superior diagnostic confidence in T1-weighted GRE imaging, in addition to reduced scan time by using more aggressive partial Fourier settings [18]. Chaika et al found that, in pancreatic MRI, a DL SR postprocessing reconstruction combined with iterative denoising outperformed standard VIBE imaging [19]. It delivered superior image quality, diminished noise and artifacts, heightened organ contrast, and improved the visibility of vessels and pancreatic ducts. Two further studies also demonstrated the improvement in image quality and lesion visibility, coupled with decreased acquisition time, by using DL SR reconstruction technique in abdominal and abdominopelvic MRI [20, 21]. Notably, compared to prior single-center retrospective studies with limited number of patients (i.e., 32–50) [18–21], our study distinguished itself with a more substantial sample size lending greater validity to the presented results and adding robustness to the conclusions. Additionally, our study facilitated the creation of a clinically applicable high-resolution imaging dataset with a 1-mm slice thickness. Furthermore, by employing an in-line reconstruction technique as opposed to the off-line approach, we significantly enhanced the clinical feasibility and efficiency. However, further validation of our findings in larger-scale multicenter prospective cohorts would be beneficial to confirm their generalizability.

In our study, DL CAIPIRINHA-VIBE delivered superior image quality compared to standard CAIPIRINHA-VIBE, on both pre-contrast and HBP images, while utilizing a reduced acquisition time (11 s vs 17 s). The observed results were largely due to the inherent benefits of DL algorithms integrated into DL CAIPIRINHA-VIBE. The primary k-space to image construction, anchored in a variational network architecture, ensured amplified noise mitigation and outstanding artifact suppression [28, 30, 36, 39]. Such enhancements made it feasible to cut down on acquisition time—a crucial step for minimizing motion artifacts in liver MRI, particularly among groups like the elderly or those with compromised breath-hold capacity. Adding to this, the SR methodology focused on refining through-plane resolution, tailored specifically for the partial Fourier acquisition in use [18–21]. This at least partly clarified the noted improvements in liver edge definition, hepatic vessel visibility, and bile duct clarity in DL CAIPIRINHA-VIBE images.

On DL CAIPIRINHA-VIBE images, the noticeable reduction in artifacts, such as respiratory motion, cardiac ghosting, and ringing, can be credited both to the decreased scan time and the artifact suppression algorithm. The scanning duration with DL CAIPIRINHA-VIBE was reduced to 11 s from the conventional 17 s, a substantial advantage in minimizing motion artifacts. The benefits stemmed not just from the variational network architecture used for the initial k-space to image reconstruction, but also from a more effective sampling scheme for spectral fat suppression incorporated in the DL CAIPIRINHA-VIBE. As current understanding suggests, trimming scan time remains the best approach to mitigate the adverse impacts of motion artifacts [10]. Nevertheless, for patients particularly susceptible to breath-holding failure, alternative methods are worth considering. Techniques such as incoherent Cartesian k-space sampling combined with motion-resolved compressed sensing reconstruction present a potentially viable option [40].

It was worth noting that DL CAIPIRINHA-VIBE exhibited a more pronounced synthetic appearance compared to standard CAIPIRINHA-VIBE on both pre-contrast and HBP images. In line with previous evaluations based on variational networks [21, 36], it is worth noting that smoothing was the main observed adversary. No change of image content was observed when compared to the conventional acquisition. This phenomenon likely arose from the strong regularization parameters in DL CAIPIRINHA-VIBE, which will enhance the perceived SNR of reconstructed images. Nevertheless, this might cause excessive smoothing of intricate structures or introduction of unfamiliar image textures, resulting in images that appeared cartoon-like or somewhat inauthentic [34, 39]. As the regularization can be tuned by the user, one aspect is that the chosen setting was considered a reasonable compromise and allowed for a significant improvement compared to the clinical standard. Future developments in network design will undoubtedly focus on optimizing image perception at a given regularization strength.

In our study, HR-DL CAIPIRINHA-VIBE notably enhanced lesion conspicuity compared with standard CAIPIRINHA-VIBE or DL CAIPIRINHA-VIBE, especially for small solid FLLs. Of note, we employed an acceleration factor of 6 for HR-DL CAIPIRINHA-VIBE as opposed to an acceleration factor of 4 for standard CAIPIRINHA-VIBE. This enhancement was achievable due to the superior noise reduction and artifact suppression capabilities of the DL algorithm compared to the parallel imaging technique. Moreover, it is important to note that the same neural networks were used in both DL and HR-DL CAIPIRINHA-VIBE protocols, ensuring consistency in our deep learning techniques. While the HR-DL CAIPIRINHA-VIBE protocol had lower SNR due to smaller voxel size and higher inherent noise, this trade-off is justified. In the HBP, signal enhancement from gadoxetic acid compensates for lower SNR, enabling effective use of higher acceleration in HR-DL for detailed imaging resolution. In our study, the improved lesion conspicuity and detection can be partly attributed to the heightened image sharpness and reduced partial volume averaging, thanks to the thinner section thickness (HR-DL vs standard, 1 mm vs 3 mm) achieved by employing DL-based image interpolation and SR algorithms. Additional factors contributing to these observations included minimized motion-related blurring and the reduction of various artifacts. As anticipated, the enhanced lesion conspicuity and reduced section thickness of HR-DL CAIPIRINHA-VIBE HBP images did not significantly affect the detection of larger lesions (≥ 2 cm). However, the subgroup analysis revealed that such improvement substantially increased the detection of small (< 2 cm) solid FLLs (the prevalence rate, 66.7% [58/87]), with the odds of detecting lesions increasing by 83% when using HR-DL CAIPIRINHA-VIBE HBP images. This improvement is clinically significant, as it can facilitate earlier disease diagnosis, precise tumor staging, and consequently, more informed therapeutic decision-making. In our institution, the DL reconstruction technique has been integrated into the standard clinical workflow for abdominal MRI studies.

Our study had several limitations. First, as a single-institution retrospective investigation, there might be inherent selection bias. Excluding patients with more than five FLLs may have also introduced a selection bias in the analysis of lesion conspicuity. Extrapolating our findings to multicenter prospective studies is warranted to substantiate their robustness and generalizability. Second, the utilization of a single MRI scanner with 3 T for our study participants suggests that our findings might not be universally applicable across different MRI scanners with various field strengths. Third, due to a paucity of histologically confirmed FLLs, a direct comparison of the diagnostic performance between standard CAIPIRINHA-VIBE and HR-DL CAIPIRINHA-VIBE was not performed. Fourth, we refrained from conducting a quantitative assessment of image quality, as it might be unreliable via conventional region-of-interest measurements of signal intensity and noise level, especially when parallel acquisition techniques or non-linear reconstruction methods like DL-based algorithms are involved [41]. Finally, the levels of interobserver agreement for several image quality parameters, such as respiratory motion artifact and liver edge sharpness, were relatively low. This suggests that certain image quality parameters could be largely influenced by reader subjectivity [34]. Future studies with more standardized imaging criteria and more rigid reading training strategies are warranted to improve the interobserver consistency.

In conclusion, compared to standard CAIPIRINHA-VIBE, DL and HR-DL CAIPIRINHA-VIBE demonstrated superior overall image quality and diminished artifacts, albeit with more synthetic appearance, on pre-contrast and HBP images, with the additional benefit of reduced acquisition time. Moreover, HR-DL CAIPIRINHA-VIBE improved the lesion conspicuity and detection of small solid FLLs on HBP images. DL and HR-DL CAIPIRINHA-VIBE hold the potential to serve as valuable alternatives in routine clinical liver MRI.

## Supplementary Information

Below is the link to the electronic supplementary material.

Supplementary file1 (PDF 775 KB)

## Abbreviations

3D: Three-dimensional
AP: Arterial phase
APHE: Arterial phase hyperenhancement
CAIPIRINHA: Controlled aliasing in parallel imaging results in higher acceleration
CS: Compressed sensing
CT: Computed tomography
DL: Deep learning
FLL: Focal liver lesion
FNH: Focal nodular hyperplasia
GRAPPA: Generalized autocalibrating partially parallel acquisition
GRE: Gradient-recalled echo
HBP: Hepatobiliary phase
HCC: Hepatocellular carcinoma
HR: High resolution
MRI: Magnetic resonance imaging
OR: Odds ratio
PAT: Parallel acquisition technique
PI: Parallel imaging
SNR: Signal-noise ratio
SR: Super-resolution
VIBE: Volumetric interpolated breath-hold examination

## Declarations

### Guarantor

The scientific guarantor of this publication is Jeong Min Lee, M.D.

### Statistics and biometry

The Medical Research Collaborating Center of Seoul National University Hospital provided assistance with the statistical analyses.

### Methodology

• retrospective

• observational study

• single-center study
