# Supplementary material for: Enhancing gadoxetic acid–enhanced liver MRI: a synergistic approach with deep learning CAIPIRINHA-VIBE and optimized fat suppression techniques
Source: Eur Radiol. 2024 Mar 16;34(10):6712–25. doi: 10.1007/s00330-024-10693-9 (PMC11399219; doi:10.1007/s00330-024-10693-9)
Supplement: Supplementary file 1 — Supplementary file1 (PDF 775 KB) [file 330_2024_10693_MOESM1_ESM.pdf]

Enhancing Gadoxetic Acid-enhanced Liver MRI: A Synergistic Approach with Deep Learning CAIPIRINHA-VIBE and Optimized Fat Suppression Techniques

ELECTRONIC SUPPLEMENTARY MATERIAL

Table S1 Details of interobserver agreement for image quality assessment of standard, DL, and HR-DL CAIPIRINHA-VIBE on pre-contrast and HBP images

Image Quality Parameter	Pre-contrast Image: Standard CAIPIRINHA-VIBE	Pre-contrast Image: DL CAIPIRINHA-VIBE	HBP Image: Standard CAIPIRINHA-VIBE	HBP Image: DL CAIPIRINHA-VIBE	HBP Image: HR-DL CAIPIRINHA-VIBE
**Interobserver Agreement between Reader 1 and 2**					
Liver edge sharpness	0.349 (0.263, 0.434)	0.149 (0.081, 0.218)	0.324 (0.248, 0.401)	0.136 (0.074, 0.199)	0.066 (0.011, 0.120)
Hepatic vessel conspicuity	0.428 (0.341, 0.515)	0.471 (0.407, 0.534)	0.177 (0.114, 0.240)	0.327 (0.275, 0.378)	0.327 (0.270, 0.384)
Bile duct conspicuity	…	…	0.380 (0.320, 0.440)	0.357 (0.297, 0.417)	0.318 (0.257, 0.379)
Respiratory motion artifact	0.106 (0.027, 0.186)	0.056 (-0.011, 0.123)	0.211 (0.143, 0.279)	0.117 (0.053, 0.182)	0.187 (0.115, 0.260)
Cardiac ghosting artifact	0.202 (0.127, 0.276)	0.283 (0.221, 0.344)	0.205 (0.130, 0.280)	0.283 (0.222, 0.343)	0.336 (0.260, 0.411)
Ringing artifact	0.732 (0.682, 0.782)	0.658 (0.604, 0.713)	0.686 (0.635, 0.736)	0.568 (0.513, 0.623)	0.659 (0.601, 0.716)
Perceived SNR	0.841 (0.799, 0.883)	0.878 (0.843, 0.914)	0.480 (0.432, 0.529)	0.369 (0.310, 0.427)	0.423 (0.362, 0.484)
Subjective Noise Level	0.698 (0.650, 0.745)	0.764 (0.718, 0.810)	0.802 (0.752, 0.851)	0.809 (0.762, 0.855)	0.624 (0.552, 0.696)
Synthetic Appearance	0.479 (0.418, 0.540)	0.437 (0.386, 0.487)	0.444 (0.382, 0.506)	0.286 (0.232, 0.340)	0.234 (0.169, 0.298)
Overall artifact level	0.856 (0.815, 0.897)	0.908 (0.878, 0.939)	0.857 (0.816, 0.898)	0.905 (0.874, 0.937)	0.708 (0.648, 0.768)
Overall image quality	0.818 (0.773, 0.862)	0.936 (0.911, 0.962)	0.830 (0.786, 0.873)	0.920 (0.891, 0.949)	0.735 (0.678, 0.792)
**Interobserver Agreement between Reader 2 and 3**					
Liver edge sharpness	0.723 (0.670, 0.777)	0.770 (0.725, 0.815)	0.746 (0.691, 0.802)	0.757 (0.708, 0.806)	0.302 (0.215, 0.390)
Hepatic vessel conspicuity	0.686 (0.634, 0.739)	0.762 (0.716, 0.807)	0.645 (0.582, 0.708)	0.705 (0.652, 0.758)	0.561 (0.486, 0.635)
Bile duct conspicuity	…	…	0.769 (0.717, 0.821)	0.743 (0.691, 0.795)	0.545 (0.475, 0.615)
Respiratory motion artifact	0.693 (0.632, 0.754)	0.631 (0.571, 0.690)	0.725 (0.660, 0.790)	0.595 (0.525, 0.665)	0.335 (0.248, 0.421)
Cardiac ghosting artifact	0.728 (0.675, 0.780)	0.814 (0.772, 0.856)	0.751 (0.694, 0.808)	0.774 (0.725, 0.823)	0.618 (0.546, 0.69)
Ringing artifact	0.801 (0.757, 0.846)	0.821 (0.778, 0.864)	0.841 (0.800, 0.882)	0.833 (0.789, 0.876)	0.613 (0.554, 0.673)
Perceived SNR	0.757 (0.709, 0.805)	0.757 (0.711, 0.803)	0.767 (0.715, 0.819)	0.743 (0.692, 0.794)	0.575 (0.508, 0.641)
Subjective Noise Level	0.749 (0.697, 0.801)	0.743 (0.695, 0.792)	0.726 (0.660, 0.791)	0.727 (0.668, 0.786)	0.507 (0.432, 0.582)
Synthetic Appearance	0.792 (0.745, 0.839)	0.825 (0.782, 0.867)	0.815 (0.767, 0.862)	0.789 (0.733, 0.845)	0.499 (0.418, 0.579)
Overall artifact level	0.782 (0.730, 0.833)	0.799 (0.753, 0.846)	0.809 (0.759, 0.858)	0.799 (0.751, 0.847)	0.689 (0.628, 0.751)
Overall image quality	0.768 (0.717, 0.819)	0.799 (0.755, 0.843)	0.768 (0.717, 0.818)	0.799 (0.753, 0.846)	0.660 (0.601, 0.718)
**Interobserver Agreement between Reader 1 and 3**					
Liver edge sharpness	0.494 (0.427, 0.561)	0.253 (0.180, 0.327)	0.472 (0.408, 0.537)	0.346 (0.271, 0.420)	0.812 (0.766, 0.857)
Hepatic vessel conspicuity	0.500 (0.430, 0.570)	0.412 (0.337, 0.487)	0.380 (0.301, 0.459)	0.330 (0.247, 0.414)	0.830 (0.787, 0.873)
Bile duct conspicuity	…	…	0.413 (0.336, 0.489)	0.373 (0.292, 0.454)	0.824 (0.778, 0.870)
Respiratory motion artifact	0.261 (0.187, 0.336)	0.268 (0.199, 0.336)	0.422 (0.350, 0.494)	0.421 (0.348, 0.494)	0.769 (0.714, 0.825)
Cardiac ghosting artifact	0.305 (0.230, 0.380)	0.285 (0.213, 0.357)	0.397 (0.330, 0.464)	0.416 (0.347, 0.485)	0.699 (0.645, 0.754)
Ringing artifact	0.475 (0.399, 0.552)	0.420 (0.337, 0.503)	0.579 (0.515, 0.644)	0.422 (0.346, 0.497)	0.763 (0.715, 0.811)

Perceived SNR	0.690 (0.634, 0.747)	0.695 (0.641, 0.748)	0.497 (0.426, 0.568)	0.523 (0.452, 0.595)	0.789 (0.737, 0.841)
Subjective Noise Level	0.736 (0.686, 0.785)	0.791 (0.744, 0.838)	0.740 (0.685, 0.795)	0.838 (0.796, 0.880)	0.776 (0.729, 0.824)
Synthetic Appearance	0.460 (0.396, 0.524)	0.459 (0.400, 0.518)	0.556 (0.502, 0.610)	0.514 (0.460, 0.568)	0.685 (0.620, 0.749)
Overall artifact level	0.801 (0.755, 0.848)	0.807 (0.763, 0.852)	0.814 (0.768, 0.860)	0.819 (0.774, 0.863)	0.763 (0.715, 0.811)
Overall image quality	0.782 (0.735, 0.829)	0.779 (0.732, 0.825)	0.754 (0.700, 0.808)	0.813 (0.768, 0.859)	0.778 (0.729, 0.827)

Data are Gwet's AC1 coefficients, with 95% confidence intervals in parentheses.

Interobserver agreement was assessed by the AC1 coefficients, as follows: <0, poor agreement; 0.01-0.20, slight agreement; 0.21-0.40, fair agreement; 0.41-0.60, moderate agreement; 0.61-0.80, substantial agreement; and 0.81-1.00, almost perfect agreement.

CAIPIRINHA, controlled aliasing in parallel imaging results in higher acceleration; DL, deep learning; HBP, hepatobiliary phase; HR, high resolution; SNR, signal noise ratio; VIBE, volumetric interpolated breath-hold examination.

Pre-contrast images

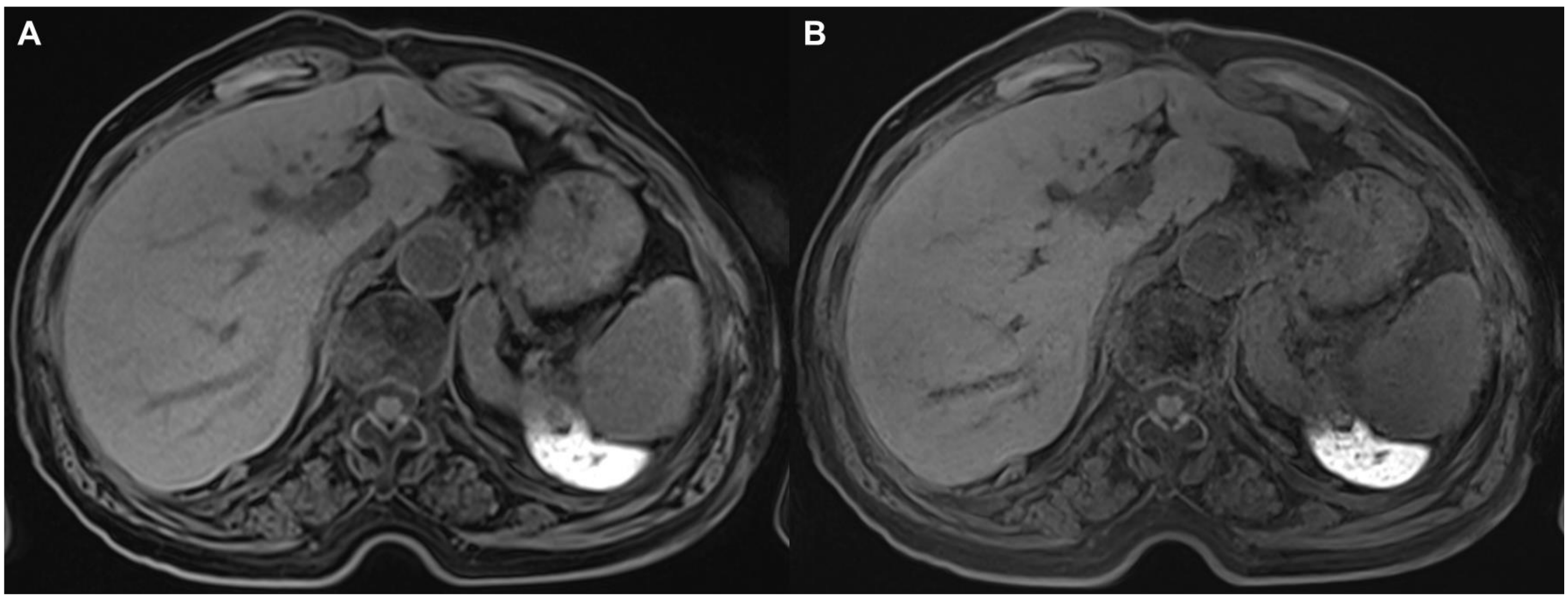

Standard CAIPIRINHA-VIBE
Mean score of subjective noise level: 3.33 points

DL CAIPIRINHA-VIBE
Mean score of subjective noise level: 2.67 points

HBP images

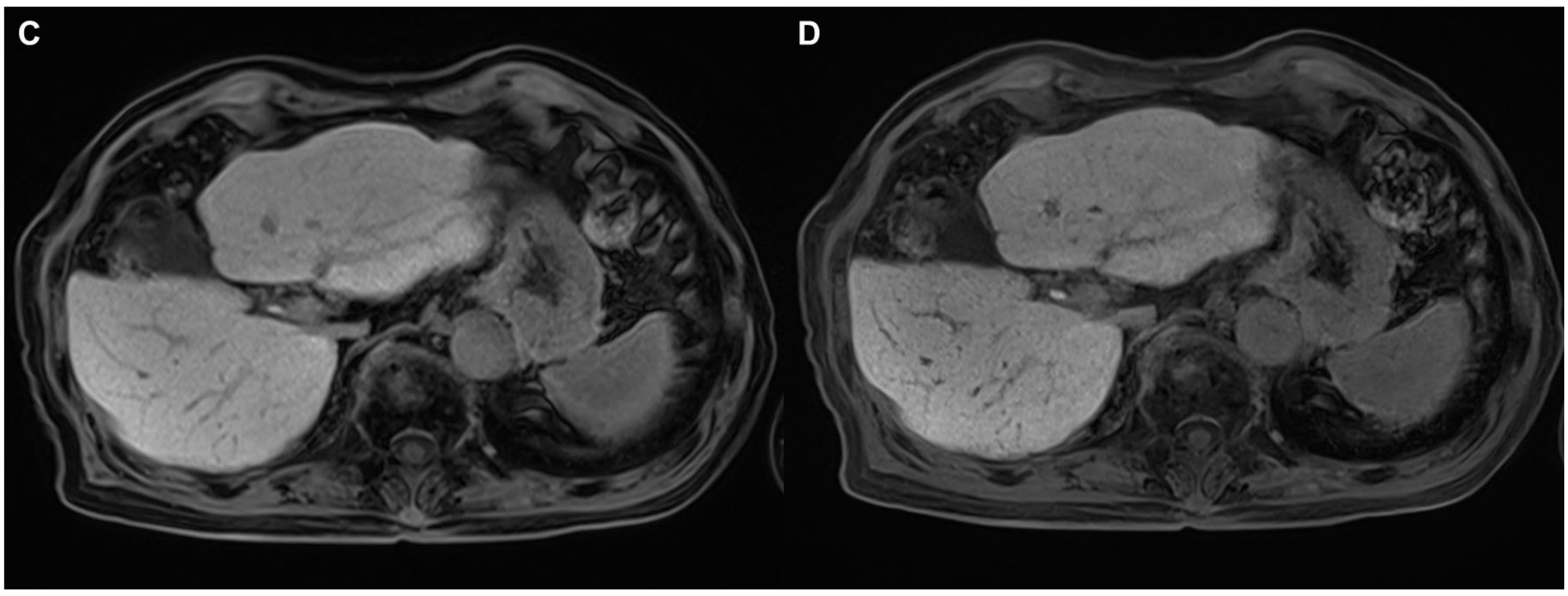

Standard CAIPIRINHA-VIBE
Mean score of subjective noise level: 3.00 points

DL CAIPIRINHA-VIBE
Mean score of subjective noise level: 2.33 points

HBP images

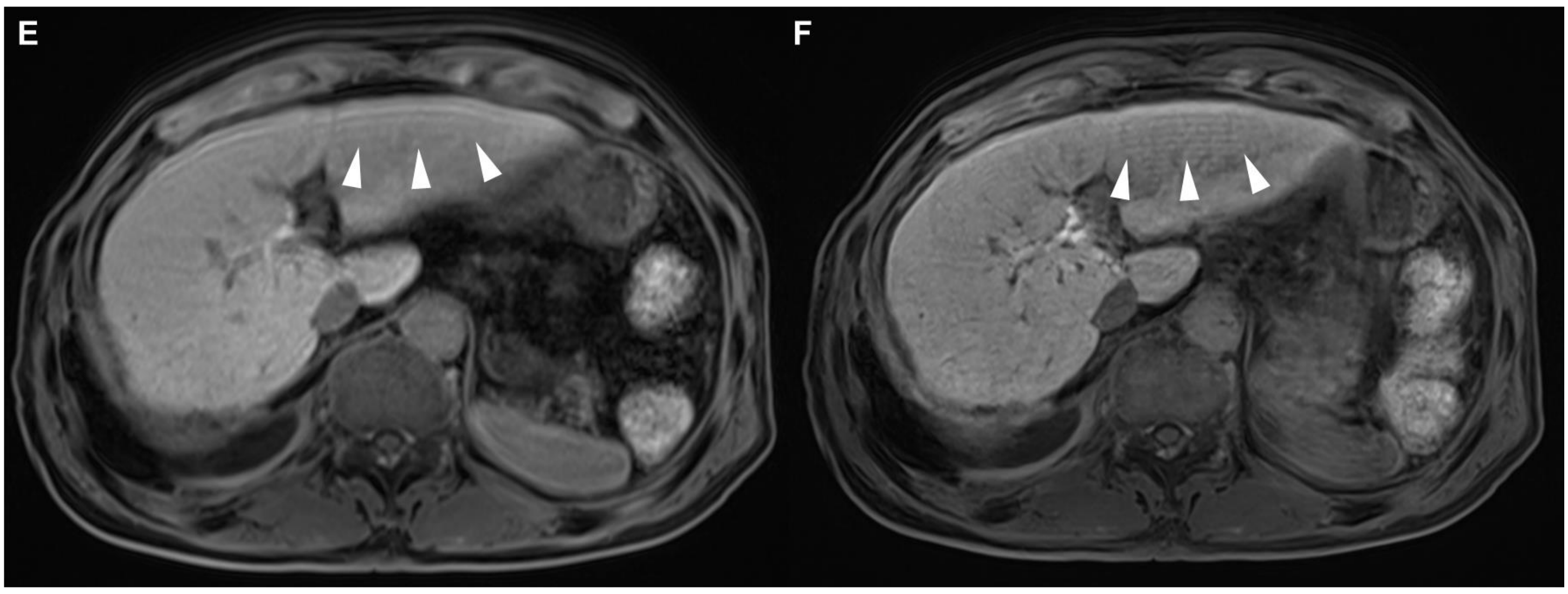

Standard CAIPIRINHA-VIBE
Mean score of respiratory motion artifact: 2.33 points

HR-DL CAIPIRINHA-VIBE
Mean score of respiratory motion artifact: 1.33 points

Figure S1 Cases with poorer image quality in DL or HR-DL CAIPIRINHA-VIBE compared to the standard CAIPIRINHA-VIBE on precontrast and HBP images. On precontrast images, the (**B**) DL CAIPIRINHA-VIBE shows lower mean score of subjective noise level than (**A**) standard CAIPIRINHA-VIBE in a 75-year-old female with HCC. On HBP images, the (**D**) DL CAIPIRINHA-VIBE shows lower mean score of subjective noise level than (**C**) standard CAIPIRINHA-VIBE in an 88-year-old male with HCC. On HBP images, the (**F**) HR-DL CAIPIRINHA-VIBE shows lower mean score of respiratory motion artifact than (**E**) standard CAIPIRINHA-VIBE in a 66-year-old male with HCC. CAIPIRINHA, controlled aliasing in parallel imaging results in higher acceleration; DL, deep learning; HBP, hepatobiliary phase; HCC, hepatocellular carcinoma; HR, high resolution; VIBE, volumetric interpolated breath-hold examination.

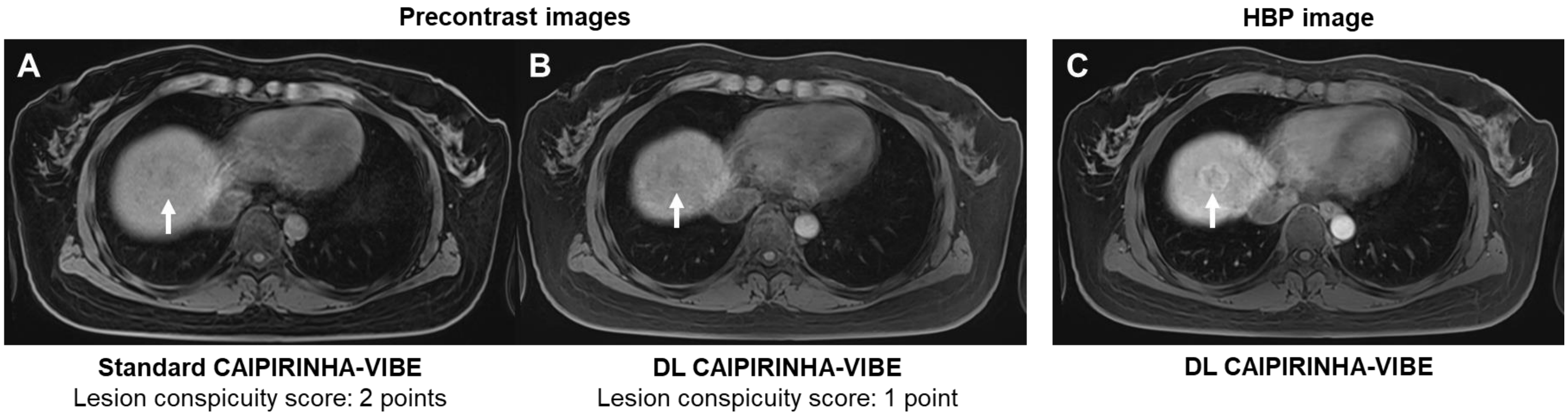

Figure S2 T1-weighted (**A, B**) precontrast and (**C**) HBP images of gadoxetic acid–enhanced MRI in a 43-year-old female with a 2.5 cm FNH (*arrows*) at segment 8 of the liver. The reviewer 1 assigned a lesion conspicuity score of 2 points in the (**A**) standard CAIPIRINHA-VIBE and of 1 point in the (**B**) DL CAIPIRINHA-VIBE on precontrast images. CAIPIRINHA, controlled aliasing in parallel imaging results in higher acceleration; DL, deep learning; FNH, focal nodular hyperplasia; HBP, hepatobiliary phase; MRI, magnetic resonance imaging; VIBE, volumetric interpolated breath-hold examination.

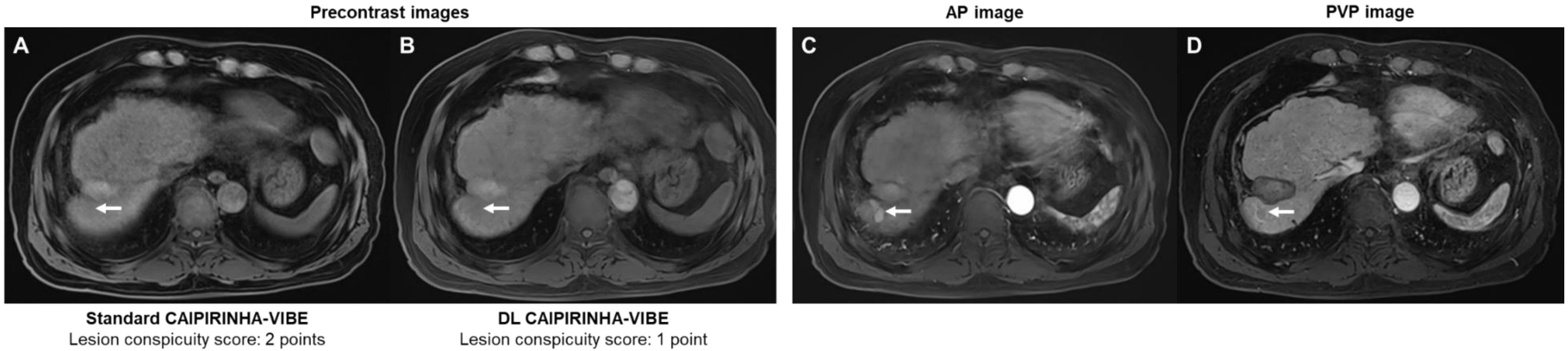

Figure S3 T1-weighted (**A, B**) precontrast, (**C**) AP and (**D**) PVP images of gadoxetic acid–enhanced MRI in a 67-year-old male with a 2.1 cm HCC (*arrows*) at segment 7 of the liver. The reviewer 2 assigned a lesion conspicuity score of 2 points in the (**A**) standard CAIPIRINHA-VIBE and of 1 point in the (**B**) DL CAIPIRINHA-VIBE on precontrast images. AP, arterial phase; CAIPIRINHA, controlled aliasing in parallel imaging results in higher acceleration; DL, deep learning; HCC, hepatocellular carcinoma; MRI, magnetic resonance imaging; PVP, portal venous phase; VIBE, volumetric interpolated breath-hold examination.

HBP images

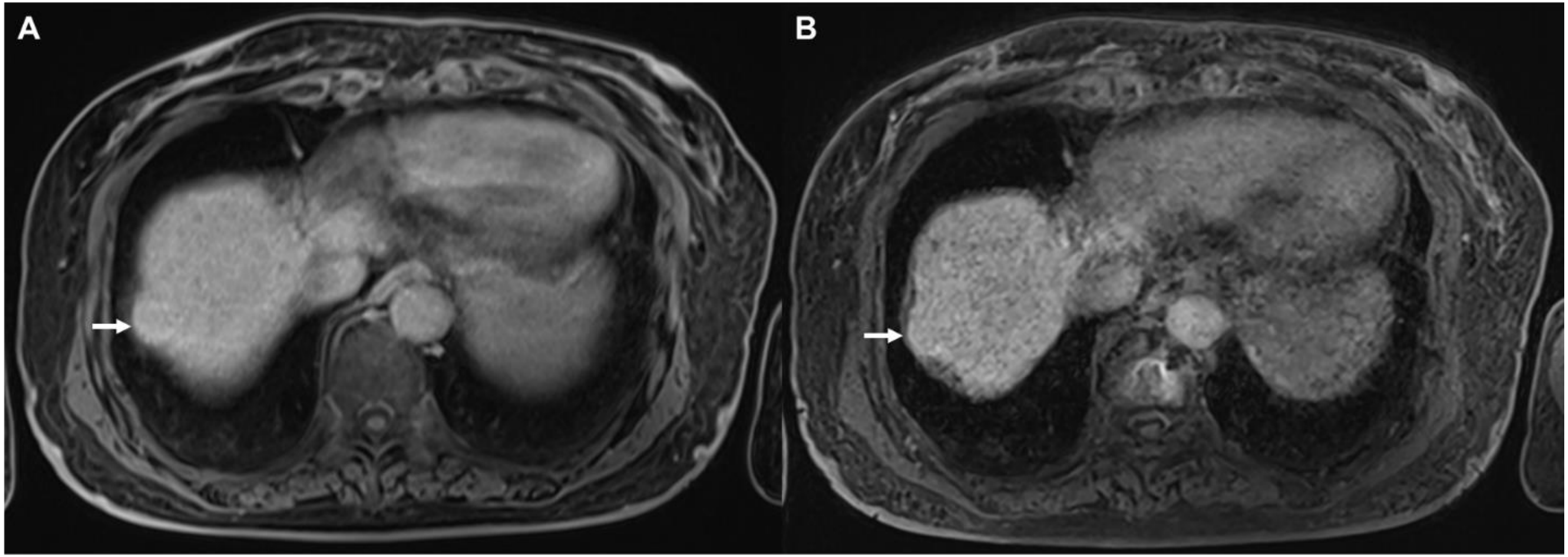

Standard CAIPIRINHA-VIBE
Lesion conspicuity score: 2 points

HR-DL CAIPIRINHA-VIBE
Lesion conspicuity score: 1 point

Figure S4 T1-weighted (**A, B**) HBP images of gadoxetic acid–enhanced MRI in a 75-year-old female with a 2.3 cm dysplastic nodule (*arrows*) at segment 8 of the liver. The reviewer 3 assigned a lesion conspicuity score of 2 points in the (**A**) standard CAIPIRINHA-VIBE and of 1 point in the (**B**) DL CAIPIRINHA-VIBE on HBP images. CAIPIRINHA, controlled aliasing in parallel imaging results in higher acceleration; DL, deep learning; HBP, hepatobiliary phase; MRI, magnetic resonance imaging; VIBE, volumetric interpolated breath-hold examination.
